# Association between oral diseases and impact on daily performance among male Saudi schoolchildren

**DOI:** 10.1002/cre2.231

**Published:** 2019-08-08

**Authors:** Abdullah Ali H Alzahrani, Eltayeb Mohammed Alhassan, Mohammad Aref Albanghali

**Affiliations:** ^1^ Dental Health Department, Faculty of Applied Medical Sciences Albaha University Albaha Saudi Arabia; ^2^ Public Health Department, Faculty of Applied Medical Sciences Albaha University Albaha Saudi Arabia

**Keywords:** children, dental status, impacts, oral health, quality of life

## Abstract

**Objective:**

Little is known about exploring the oral health related quality of life (OHRQoL) of school children in Saudi Arabia. The importance of examining this topic has centred on its ability to measure associations between the oral symptoms, impacts and clinical measures. This study aims to explore associations between the (OHRQoL) based on the children's Oral Impacts on Daily Performance (Child‐OIDP) index and oral diseases of Saudi school children living in Albaha region.

**Materials and methods:**

A cross‐sectional study was conducted between November 2017 to January 2018 using two‐stage randomised sampling technique. The oral diseases and impacts were examined using the World Health Organization's (1997) guidelines and the Child‐OIDP index, respectively. Data analysis was conducted using the SPSS software version 20.0. Chi‐squared, bivariate and multivariate logistic regression tests were used, as appropriate.

**Results:**

In total 349 Saudi male school children, 12 to 15 years old, were recruited. The Child‐OIDPrate was significantly high (75.1%). The participants reported minor, moderate and major (severe) oral impacts (29.4%, 50%, and 20.6% respectively). The overall mean of the Child‐OIDP score was 2.15±1.40.

**Conclusions:**

The high overall Child‐OIDP score was in accordance with the participants' dental status. Further research should focus on improving knowledge and implementing education programmes to advance the community's oral diseases and practices.

**Why this paper is important to paediatric dentists?**

- This study uncovers that the high overall Child‐OIDP score was in accordance with the high prevalence of dental caries among the studied population. Hence, paediatric dentists may require to take into consideration the associations between the oral symptoms, impacts, and clinical indicators when treating children.
- Oral impacts on daily performances (OIDP) were commonly reported on eating and enjoying food, cleaning teeth, and smiling. Consequently, paediatric dentists should not omit assessing those impacts, besides providing proper oral health guidance and dental care for children.
- Future research may focus on improving children knowledge through school visits, implementing effective oral health education and promoting programmes to advance the oral health status, attitude, and practices of children.

## 1 INTRODUCTION

The concept of the oral health‐related quality of life (OHRQoL) has been described as “a multidimensional construct that includes a subjective evaluation of the individual's oral health, functional well‐being, emotional well‐being, expectations and satisfaction with care, and sense of self” (Sischo & Broder, 2011). The World Health Organization has considered this concept as an essential part of the Global Oral Health Programme and as a basic measure of an individual's overall health and well‐being (Petersen, 2003). The OHRQoL has significant implications for both dental research and clinical dentistry, which can be seen in the recent tendency towards exploring and evaluating the OHRQoL, particularly among children, worldwide (Amalia, Schaub, Stewart, Widyanti, & Groothoff, 2017; Barbosa, Gavião, Leme, & Castelo, 2016; Sun, Wong, & McGrath, 2018).

The evaluation of the OHRQoL describes a shift from traditional clinical dental criteria to specific evaluations that centre on an individual's physical functions, emotions, and social experiences when determining suitable therapy goals and outcomes (Sischo & Broder, 2011). Examining the OHRQoL has uncovered the difference between evaluations of perceived and normative dental treatment needs. Moreover, clinical indicators of oral health status were significantly related to OHRQoL measures (Tsakos, Marcenes, & Sheiham, 2004). This highlights the importance of measuring the OHRQoL in any community. Nevertheless, evidence has demonstrated that oral health status assessments have most commonly employed evaluations of clinical dental measures only. However, the resulting data would be more valuable if OHRQoL scales were employed in conjunction with these clinical measures (Gherunpong, Tsakos, & Sheiham, 2006).

There are several measures that can be used for examining the OHRQoL, including the social impacts of dental disease, dental impact profile, child oral health impact profile, subjective oral health status indicators and children's version of the oral impacts on daily performance (Child‐OIDP) (Allen, 2003). However, the Child‐OIDP is focused on an unambiguous conceptual framework that has the ability to capture the more intermediate and proximal impacts of disabilities and handicaps, such as dissatisfaction with appearance, functional limitations, discomfort, and pain (Jokovic et al., 2002; World Health Organisation, 1980). For the Child‐ODIP, this is one distinctive advantage when compared with the other OHRQoL measures, in addition to its reliability and validity across different countries, including, but not limited to, Brazil (Castro et al., 2008), Sweden (Ostberg, Andersson, & Hakeberg, 2008), the United Kingdom (Yusuf, Gherunpong, Sheiham, & Tsakos, 2006), China (Hongxing, List, Nilsson, Johansson, & Astrøm, 2014), India (Agrawal, Pushpanjali, & Garg, 2013), Sudan (Nurelhuda, Ahmed, Trovik, & Åstrøm, 2010), and Morocco (Lazrak, Bourzgui, Serhier, Diouny, & Othmani, 2017a). It was for this reason that the Child‐OIDP scale was used in the current study.

The OHRQoL plays a significant role in understanding a subject's individual evaluation of his or her dental care by describing his or her comfort levels while eating, sleeping, smiling, and engaging in social interactions, as well as his or her satisfaction and self‐esteem with respect to oral health (Genderson, Sischo, Markowitz, Fine, & Broder, 2013). However, there have been no previous studies that have assessed the OHRQoL of Saudi children using the Child‐OIDP scale, particularly in the Albaha province. Therefore, the aim of this study was to examine the associations between the OHRQoL based on the Child‐OIDP index and the different oral diseases among Saudi schoolchildren living in the Albaha region of Saudi Arabia.

## 2 MATERIALS AND METHODS

### 2.1 Settings and participants

A cross‐sectional descriptive study was conducted among male Saudi schoolchildren, 12–15 years old, from three different schools across three dissimilar areas (Alatawelah, Albaha, and Alaquiq) in the Albaha province of Saudi Arabia. Study information sheets and informed consent forms were sent to the parents of these schoolchildren between November 2017 and January 2018. Those children who were physically and mentally fit for this study, and whose parents provided written (informed) consent for them to participate, were included in this research. Those children with histories of antibiotic therapy and/or systemic diseases during the previous 3 months were excluded. Similarly, female schoolchildren were excluded from the study sample mainly due to the cultural challenges encountered when attempting to access females of this age group in their schools, because the researchers who conducted this study were men. The subjects were then classified according to three different age groups: 12–13, 14, and 15 years old. The participants were interviewed to determine their OHRQoL using the Child‐OIDP index, and each of them underwent clinical oral examinations to evaluate their overall oral health status.

### 2.2 Sampling

A two‐stage randomised sampling method was employed between February 2018 and April 2018 to determine the study sample. During the first stage, the statistical information concerning the details of the male intermediate schools and total number of students in the region was obtained from the local education authority (the Albaha branch of the Saudi Ministry of Education). At this stage, 59 male intermediate schools and 6,188 enrolled students were identified across the three territories (Alatawelah, Albaha, and Alaquiq) in the Albaha province. During the second stage, one school was randomly selected from each of the three previous territories, and then some students were randomly selected for participation in this study.

Because the total number of students enrolled in male intermediate schools in Albaha province was (*n* = 6,188), and for a confidence level of 95% and marginal error of (±0.05), the calculated sample size for this study was approximately 362 participants. However, we proposed to recruit 400 participants to avoid any refusal in participation and improve the recruitment response rate.

### 2.3 Children oral examination

The study subjects were examined clinically in order to determine their oral diseases using the basic methods developed by the World Health Organization (1997) (World Health Organization 1997). The oral examinations were performed using disposable oral examination kits consisting of a community periodontal index probe, sharp probe (Number 4), mouth mirror, face mask, tweezers, gauze, cotton, and gloves.

### 2.4 Child‐OIDP Questionnaire

The data were gathered using the translated Arabic version of the Child‐OIDP questionnaire developed by Gherunpong et al. (Gherunpong, Tsakos, & Sheiham, 2004a). The process of translation and adaption of the Child‐OIDP questionnaire involved six key phases: (1) assigning the specialist panel, which consisted of two dental specialists, one public health specialist, and a native Arabic‐speaking translator from accredited English–Arabic Translation Centre. The translator was unaware of the aim and concept of the study. (2) The specialist panel translated the Child‐OIDP questionnaire from English to Arabic for establishing the first Arabic version. (3) Piloting the first Arabic version in a group of children (10%) as recommended by the WHO. (4) The panel meet to produce the second version of the Arabic Child‐OIDP version after piloting. (5) The panel back translated the second version of the Arabic Child‐OIDP to English. (6) The panel reassessed all versions of the Child‐OIDP and collectively compiled a single Arabic version of Child‐OIDP.

The questionnaire consisted of two key sections. The first section focused mainly on the sociodemographic data, such as the age, education level, and geographical location. The second section centred on collecting the data related to the Child‐OIDP itself. The Child‐OIDP evaluates the psychological, physical, and social aspects of eight daily performance factors (Gherunpong, Tsakos, & Sheiham, 2004b) (Table 1). In order to reduce the risk of bias as much as possible and to prevent the manipulation of the clinical examinations based on prior knowledge of the Child‐OIDP score, the examiners were asked to record the clinical examination findings separate from the Child‐OIDP results.

**Table 1:** The children's version of the oral impacts on daily performance index factors

	Performance factor
1	Eating and enjoying food
2	Speaking and pronouncing clearly (speaking)
3	Cleaning teeth
4	Smiling, laughing, and showing teeth without embarrassment (smiling)
5	Relaxing and sleeping
6	Maintaining a useful emotional state without being irritable (emotional stability)
7	Carrying out major school work or social roles (school work)
8	Enjoying social contact with people (social contact)

The participants were asked to define their perceived oral issues over the past 3 months. If a subject reported an oral impact on any daily performance factor, the severity was recorded using a 3‐point ordinal scale (1 = “minor severe effect,” 2 = “moderate severe effect,” and 3 = “major severe effect”). However, if a participant experienced no impact, a score of zero was used. While the frequency of the impacts was recorded using a 3‐point ordinal scale (1 = “once or twice a month,” 2 = “three times a month, or once or twice a week,” 3 = “three or more times a week”). A zero score for frequency was assigned if the participant had never affected in the past 3 months (Gherunpong et al., 2004b). The performance factor scores were estimated by multiplying the frequency scores and the severity scores. The overall score of the Child‐OIDP was the sum of the eight performance factor scores multiplied by 100 and divided by the maximum possible score (72) (Gherunpong et al., 2004b).

### 2.5 Pilot assessment and psychometric properties of the index

Based on the recommendations of the World Health Organization (1997) (World Health Organization 1997), a pilot assessment of 10% of the randomly chosen cases (i.e., 40 participants) across the three different areas (Alatawelah, Albaha, and Alaquiq) was examined and re‐examined after 1 week by an independent examiner in order to gauge the intraexaminer agreement and consistency with regard to the Child‐OIDP evaluation. The interrater reliability was measured using weighted Cohen's kappa. The weighted kappa values for the Child‐OIDP items were 0.86, 0.92, and 0.88, respectively, for eating, smiling, and school work, whereas the weighted kappa values for the other items (speaking, cleaning, relaxing, emotional stability, and social contact) were all 0.89, indicating great agreement and consistency between the outcomes of the two examiners. Moreover, the psychometric properties of the Child‐OIDP index was evaluated. The internal consistency of the Child‐OIDP index indicated strong consistency with estimated standardised Cronbach's *α* = .784. Table 2 illustrates inter‐item correlation coefficients for the Child‐OIDP index, whereas corrected item‐total correlation of the Child‐OIDP performances and Cronbach's alpha coefficients was described in Table 3.

**Table 2:** Inter‐item correlation coefficients for the Child‐OIDP index

Performance	Eating	Speaking	Cleaning teeth	Smiling	Relaxing and sleeping	Emotional stability	School work	Social contact
Eating	1.000	—	—	—	—	—	—	—
Speaking	0.126	1.000	—	—	—	—	—	—
Cleaning teeth	0.257	0.209	1.000	—	—	—	—	—
Smiling	0.423	0.101	0.263	1.000	—	—	—	—
Relaxing and sleeping	0.287	0.257	0.246	0.242	1.000	—	—	—
Emotional stability	0.199	0.312	0.324	0.387	0.297	1.000	—	—
School work	0.266	0.157	0.253	0.143	0.356	0.211	1.000	—
Social contact	0.197	0.198	0.372	0.312	0.386	0.321	0.342	1.000

**Table 3:** Corrected item‐total correlation of the Child‐OIDP performances and Cronbach's alpha coefficients

Performance	Corrected item‐total correlation	Cronbach's alpha if item deleted
Eating	0.431	0.762
Speaking	0.376	0.774
Cleaning teeth	0.434	0.753
Smiling	0.571	0.751
Relaxing and sleeping	0.492	0.742
Emotional stability	0.393	0.733
School work	0.531	0.724
Social contact	0.587	0.736
Alpha value	—	0.762
Standardised items alpha	—	0.784

### 2.6 Statistical analysis

The data analysis was conducted using the Statistical Package for the Social Sciences version 2.0. The following independent variables were basically examined as described by the World Health Organisation (1997) (World Health Organisation, 1997) and based on the presence or absence of those clinical independent variables: toothache, sensitive teeth, tooth decay (caries or cavities), tooth spacing (due to an unerupted permanent tooth or teeth), fractured permanent tooth (or teeth), tooth colour, tooth shape or size, tooth position (e.g., crooked or projecting gap), bleeding gums, swollen gums, calculus, oral ulcers, bad breath, erupting permanent tooth (or teeth), and missing permanent tooth (or teeth). The dependent variables were the eight Child‐OIDP daily performance factors (Table 1).

The exact number of observations and their associated percentages were used for the descriptive analysis, and the mean and standard deviation were reported, as appropriate. The chi‐squared test was used to evaluate the associations between the dichotomised oral diseases and the oral impacts on the daily performance factors. A multivariate logistic regression model was used to assess associations between the Child‐OIDP daily performance factors (dependent variables) and the oral diseases (independent variables). Moreover, obtained odds ratio (OR) with associated *p* value were adjusted for all of the 15 oral diseases. Nagelkerke *R* ^2^ value was employed to examine fit of the models.

## 3 RESULTS

### 3.1 Participants characteristics

**Figure 1:**
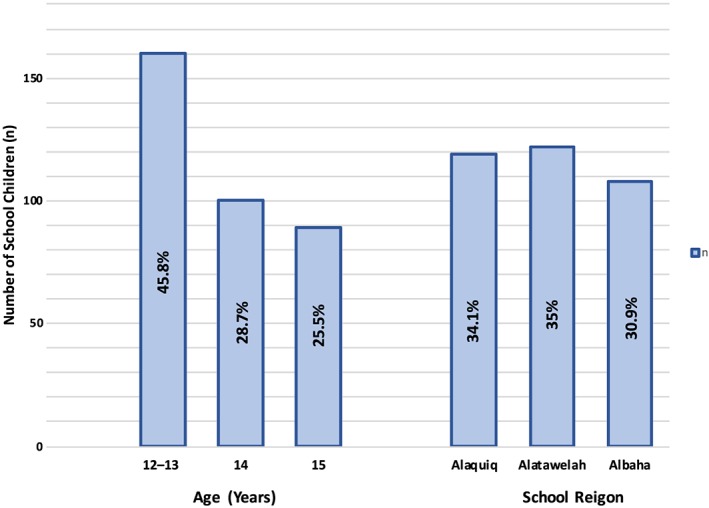
Sociodemographic characteristics of subjects according to school region and age group (*n* = 349)

From three different schools in the Albaha province, a total of 400 male Saudi schoolchildren, 12–15 years old, were asked to participate in this study. Twenty‐nine of the students were absent on either the day of the interview or the day of the clinical examination. Another 22 students refused to participate. Therefore, 349 schoolchildren were finally included in the current study (i.e., a response rate of 87.25%). The sociodemographic characteristics of those schoolchildren were recorded according to the school region and age group (Figure 1).

### 3.2 Overall oral health status of the study population

Of the 349 subjects, the dental caries, gingival bleeding, and visible plaque prevalences were 79% (*n* = 276), 83% (*n* = 289), and 91% (*n* = 317), respectively. The Decayed, Missing or Filled Teeth (DMFT) mean score was 0.49 ± 0.61, and the means of the gingival bleeding and visible plaque indices were 1.10 ± 0.88 and 1.38 ± 0.70, respectively. There were no statistically significant differences in the DMFT, gingival bleeding, and visible plaque scores between the different age groups and regions.

### 3.3 Prevalence and severity of the oral impacts

The prevalence of the oral impacts on the children's daily performance factors during the previous 3 months was significantly high (75.1%). Eating and enjoying food was the most frequently impacted daily performance factor (54.4%), followed by cleaning teeth (46.7%). The least impacted daily performance factors were relaxing and sleeping and social contact (3.2% and 3.4%, respectively). The overall mean Child‐OIDP score was 2.15 ± 1.40. Those participants reporting oral impacts described different severities for them. “Minor severe effects” were reported by 29.4% of the participants, “moderate severe effects” were reported by 50%, and “major severe effects” were reported by 20.6%. Table 4 illustrates the oral impact prevalences, Child‐OIDP scores, and severity scores.

**Table 4:** The oral impact prevalences, children's version of the oral impacts on daily performance (Child‐OIDP) scores and severity scores

	Overall	Eating and enjoying food	Speaking	Cleaning teeth	Smiling	Relaxing and sleeping	Emotional stability	School work	Social contact
Oral impact prevalence n (%)	262 (75.1)	190 (54.4)	15 (4.3)	163 (46.7)	40 (11.5)	11 (3.2)	4 (1.1)	43 (12.3)	12 (3.4)
Mean Child‐OIDP score	2.15 (1.40)	2.25 (1.52)	1.85 (1.04)	2.20 (1.40)	2.43 (1.66)	2.22 (1.92)	2.08 (1.15)	2.09 (1.50)	1.71 (1.43)
Oral impact severities among the participants reporting impacts *n* (%)									
Minor severe effect	77 (29.4)	92 (48.4)	12 (80)	26 (16)	28 (70)	2 (18.2)	2 (50)	27 (62.8)	8 (66.7)
Moderate severe effect	131 (50.0)	69 (36.3)	2 (13.3)	113 (69.3)	8 (20)	7 (63.6)	2 (50)	14 (32.6)	3 (25)
Major severe effect	54 (20.6)	29 (15.3)	1 (6.7)	24 (14.7)	4 (10)	2 (18.2)	0 (0)	2 (4.6)	1 (8.3)

### 3.4 Relationships between the oral diseases and the oral impact scores

The results of this study revealed significant associations between the oral impacts on the daily performance factors and the school location (*p* = .012), toothache (*p* < .0001), sensitive teeth (*p* < .0001), dental caries (*p* < .0001), tooth position (*p* = .0305), bleeding gums (*p* < .0001), swollen gums (*p* = .022), and missing permanent teeth (*p* = .004). However, no statistically significant differences were observed between the oral impacts on the daily performance factors and the age, tooth spacing, fractured permanent tooth (or teeth), tooth colour, tooth shape or size, calculus, oral ulcers, bad breath, and erupting permanent tooth (or teeth). Table 5 shows the analysis of the associations between the oral diseases and the oral impacts on the daily performance factors.

**Table 5:** Analysis of the associations between the oral diseases and the oral impacts on the daily performance factors (*n* = 349)

Oral clinical indicator		No oral impact (*n* = 87) *n* (%)	One or more oral impact (*n* = 262) *n* (%)	Chi‐squared value	*p* value
Age (years)	12–13	44 (50.5)	116 (44.3)	2.64	.267
	14	19 (22)	81 (30.9)		
	15	24 (27.5)	65 (24.8)		
School location	Alaquiq	40 (46)	79 (30.1)	8.82	.012
	Alatawelah	29 (33.3)	93 (35.5)		
	Albaha	18 (20.7)	90 (34.4)		
Toothache	Absent	83 (95.4)	164 (62.6)	33.98	<.0001
	Present	4 (4.6)	98 (37.4)		
Sensitive teeth	Absent	78 (89.7)	162 (61.8)	23.54	<.0001
	Present	9 (10.3)	100 (38.2)		
Tooth decay	Absent	65 (74.7)	119 (45.8)	22.58	<.0001
	Present	22 (25.3)	143 (54.2)		
Tooth spacing	Absent	76 (87.4)	223 (85.1)	0.26	.725
	Present	11 (22.6)	39 (14.9)		
Fractured permanent tooth	Absent	83 (95.4)	242 (92.4)	0.94	.464
	Present	4 (4.6)	20 (7.6)		
Tooth colour	Absent	81 (93.1)	250 (95.4)	0.716	.406
	Present	6 (6.9)	12 (4.6)		
Shape or size of tooth	Absent	86 (98.9)	252 (96.2)	1.52	.304
	Present	1 (1.1)	10 (3.8)		
Tooth position	Absent	77 (88.5)	219 (83.6)	1.226	.0305
	Present	10 (11.5)	43 (16.4)		
Bleeding gums	Absent	65 (74.7)	88 (33.6)	44.87	<.0001
	Present	22 (25.3)	174 (66.4)		
Swollen gums	Absent	81 (93.1)	217 (82.8)	5.53	.022
	Present	6 (6.9)	45 (17.2)		
Calculus	Absent	80 (92)	229 (87.4)	1.33	.333
	Present	7 (8)	33 (22.6)		
Oral ulcers	Absent	87 (100)	260 (99.2)	0.666	.414
	Present	0 (0)	2 (0.8)		
Bad breath	Absent	85 (97.7)	252 (96.2)	0.452	.501
	Present	2 (2.3)	10 (3.8)		
Erupting permanent tooth	Absent	78 (89.7)	245 (93.5)	1.409	.243
	Present	9 (10.3)	17 (6.5)		
Missing permanent tooth	Absent	82 (94.3)	260 (99.2)	8.253	.004
	Present	5 (5.7)	2 (0.8)		

All of the independent variables with significant relationships of more than one oral impacts were examined, and they are summarised in Table 5. A multivariate logistic regression of the associations between the Child‐OIDP scores (dependent variables) and the oral diseases (independent variables) was conducted. Three significant variables with more than one oral impact were recognised: toothache (*OR* = 18.82, 95% confidence interval, CI [6.44, 55.02], *p* < .0001), sensitive teeth (*OR* = 8.62, 95% CI [3.93, 18.91], *p* < .0001), and bleeding gums (*OR* = 5.02, 95% CI [2.72, 9.24], *p* < .0001).

The oral impacts on eating and enjoying food, cleaning teeth, and smiling were more likely to be influenced by the presence of dental caries and bleeding gums. The oral impacts on school work and relaxing and sleeping were more likely to be seen in the participants with toothaches and bleeding gums. Table 6 shows the distribution of the subjects based on their oral diseases and the oral impacts on their daily performance factors.

**Table 6:** Distribution of schoolchildren based on their oral diseases and the impacts on their daily performance factors (*n* = 349)

Oral health indicator			Daily performance factors							
			Eating and enjoying food	Speaking	Cleaning teeth	Smiling	Relaxing and sleeping	Emotional stability	School work	Social contact
			*n* (%)	*n* (%)	*n* (%)	*n* (%)	*n* (%)	*n* (%)	*n* (%)	*n* (%)
Toothache	Absent	247 (70.8)	104 (54.7)	8 (53.3)	107 (65.6)	21 (52.5)	4 (36.4)	4 (100)	18 (41.9)	6 (50)
	Present	102 (29.2)	86 (45.3)	7 (46.7)	56 (34.4)	19 (47.5)	7 (63.6)	0 (0)	25 (58.1)	6 (50)
Sensitive teeth	Absent	240 (68.8)	112 (58.9)	9 (60)	102 (62.6)	25 (62.5)	9 (81.8)	3 (75)	26 (60.5)	9 (75)
	Present	109 (31.2)	78 (41.1)	6 (40)	61 (37.4)	15 (37.5)	2 (18.2)	1 (25)	17 (39.5)	3 (25)
Tooth decay	Absent	184 (52.7)	73 (38.4)	8 (53.3)	77 (47.2)	19 (47.5)	4 (36.4)	4 (100)	8 (18.6)	6 (50)
	Present	165 (47.3)	117 (61.6)	7 (46.7)	86 (52.8)	21 (52.5)	7 (63.6)	0 (0)	35 (81.4)	6 (50)
Tooth spacing	Absent	299 (85.7)	165 (86.8)	14 (93.3)	140 (85.9)	30 (75)	9 (81.8)	3 (75)	34 (79.1)	9 (75)
	Present	50 (14.3)	25 (13.2)	1 (0.7)	23 (14.1)	10 (25)	2 (18.2)	1 (25)	9 (20.9)	3 (25)
Fractured permanent tooth	Absent	325 (93.1)	175 (92.1)	10 (66.7)	153 (93.9)	32 (80)	7 (63.6)	3 (75)	41 (95.2)	12 (100)
	Present	24 (6.9)	15 (7.9)	5 (33.3)	10 (6.1)	8 (20)	4 (36.4)	1 (25)	2 (4.7)	0 (0)
Tooth colour	Absent	331 (94.8)	181 (95.3)	15 (100)	154 (94.5)	35 (87.5)	11 (100)	4 (100)	40 (93)	10 (83.3)
	Present	18 (5.2)	9 (4.7)	0 (0)	9 (5.5)	5 (12.5)	0	0 (0)	3 (7)	2 (16.7)
Shape or size of tooth	Absent	338 (96.8)	184 (96.8)	13 (86.7)	154 (94.5)	34 (85)	11 (100)	4 (100)	41 (95.2)	10 (83.3)
	Present	11 (3.2)	6 (3.2)	2 (13.3)	9 (5.5)	6 (15)	0 (0)	0 (0)	2 (4.7)	2 (16.7)
Tooth position	Absent	296 (84.8)	160 (84.2)	10 (66.7)	133 (81.6)	29 (72.5)	9 (81.8)	1 (25)	35 (81.4)	8 (66.7)
	Present	53 (15.2)	30 (15.8)	5 (33.3)	30 (18.4)	11 (27.5)	2 (18.2)	3 (75)	8 (18.6)	4 (33.3)
Bleeding gums	Absent	153 (43.8)	66 (34.7)	8 (53.3)	31 (19)	11 (27.5)	4 (36.4)	2 (50)	9 (20.9)	4 (33.3)
	Present	196 (56.2)	124 (65.3)	7 (46.7)	132 (81)	29 (72.5)	7 (63.6)	2 (50)	34 (79.1)	8 (66.7)
Swollen gums	Absent	298 (85.4)	166 (87.4)	13 (86.7)	127 (77.9)	32 (80)	8 (72.7)	2 (50)	32 (74.4)	10 (83.3)
	Present	51 (14.6)	24 (12.6)	2 (13.3)	36 (22.1)	8 (20)	3 (27.3)	2 (50)	11 (25.6)	2 (16.7)
Calculus	Absent	309 (88.5)	161 (84.7)	12 (80)	139 (85.3)	36 (90)	10 (90.9)	4 (100)	32 (74.4)	10 (83.3)
	Present	40 (11.5)	29 (15.3)	3 (20)	24 (14.7)	4 (10)	1 (9.1)	0 (0)	11 (25.6)	2 (16.7)
Oral ulcers	Absent	347 (99.4)	188 (98.9)	15 (100)	161 (98.8)	40 (100)	11 (100)	4 (100)	43 (100)	12 (100)
	Present	2 (0.6)	2 (1.1)	0 (0)	2 (1.2)	0 (0)	0 (0)	0 (0)	0 (0)	0 (0)
Bad breath	Absent	337 (96.6)	184 (96.8)	15 (100)	156 (95.7)	37 (92.5)	11 (100)	4 (100)	38 (88.4)	9 (75)
	Present	12 (3.4)	6 (3.2)	0 (0)	7 (4.3)	3 (7.5)	0 (0)	0 (0)	5 (11.6)	3 (25)
Erupting permanent tooth	Absent	323 (92.6)	183 (96.3)	15 (100)	149 (91.4)	36 (90)	11 (100)	3 (75)	39 (90.7)	9 (75)
	Present	26 (7.4)	7 (3.7)	0 (0)	14 (8.6)	4 (10)	0 (0)	1 (25)	4 (9.3)	3 (25)
Missing permanent tooth	Absent	342 (98)	190 (100)	15 (100)	161 (98.8)	38 (95)	11 (100)	4 (100)	43 (100)	12 (100)
	Present	7 (2)	0 (0)	0 (0)	2 (1.2)	2 (5)	0 (0)	0 (0)	0 (0)	0 (0)

Those subjects who reported toothaches were more likely to report significant oral impacts on eating and enjoying food (*OR* = 11.5, 95% CI [5.58, 23.71], *p* < .0001), speaking (*OR* = 7.3, 95% CI [1.38, 38,82], *p* = .019), smiling (*OR* = 3.8, 95% CI [1.46, 10.12], *p* = .006), relaxing and sleeping (*OR* = 5.7, 95% CI [1.04, 31.64], *p* = .044), and school work (*OR* = 5.2, 95% CI [2.24, 11.94], *p* < .0001) than those without toothaches. Likewise, those participants with sensitive teeth were more significantly orally impacted with regard to eating and enjoying food (*OR* = 5.9, 95% CI [3.22, 11.15], *p* < .0001), speaking (*OR* = 5.6, 95% CI [1.28, 24.57], *p* = .022), cleaning teeth (*OR* = 1.6, 95% CI [0.94, 2.97], *p* = .046), and school work (*OR* = 2.7, 95% CI [1.18, 6.25], *p* = .018) than those without sensitive teeth. Table 7 shows the multivariate logistic regression analysis of the associations between the oral diseases and the oral impacts on the daily performance factors.

**Table 7:** Multivariate logistic regression analysis of the relationships between the oral impacts on the daily performance factors and the oral health indicators (*n* = 349)

Oral health indicators	Daily performance factors							
	Eating and enjoying food	Speaking	Cleaning teeth	Smiling	Relaxing and sleeping	Emotional stability	School work	Social contact
Toothache	11.502*	7.336*	1.570	3.848*	5.758*	4.457	5.180*	2.398
Sensitive teeth	5.999*	5.612*	1.680*	2.272	0.513	2.033	2.725*	0.697
Tooth decay	1.963*	0.669	0.932	0.833	0.808	0.000	1.291	0.580
Tooth spacing	1.992	1.037	0.828	2.214	3.974	3.862	2.957*	0.657
Fractured permanent tooth	1.600	6.926*	0.830	8.062*	26.489*	4.489	0.506	0.000
Tooth colour	0.708	0.000	1.041	2.886	0.000	0.000	0.332	0.420
Shape or size of tooth	0.860	9.856	10.099*	18.577*	0.000	0.000	1.045	4.511
Tooth position	1.545	3.493	1.262	1.417	1.157	3.067	0.908	2.545
Bleeding gums	1.627	0.486	7.882*	3.079*	1.476	0.000	2.023	1.097
Swollen gums	0.337*	0.553	2.130*	2.153	3.636	3.877	1.439	1.340
Calculus	6.533	4.496	0.741	0.193*	0.201	0.009	3.089	0.342
Oral ulcers	3.569	0.000	1.668	0.000	0.000	6.618	0.000	4.199
Bad breath	0.361	0.000	0.693	0.647	0.000	0.004	4.133	12.522*
Erupting permanent tooth	0.114	0.000	1.395	0.468	0.000	3.448	0.494	2.695
Missing permanent tooth	0.000	0.000	1.321	7.592*	0.000	0.154	0.000	0.000
Nagelkerke *R* ^2^	0.466	0.285	0.343	0.275	0.288	0.615	0.243	0.183

* Adjusted odds ratio values: *p* values <.05 were statistically significant.

## 4 DISCUSSION

Combining the clinical dental measures, such as the DMFT score, and the sociodental measures, such as the Child‐OIDP scores, might enable a broader approach to dental care (Gherunpong et al., 2006; Leao & Sheiham, 1996). However, there is lack of such studies in the dental literature, especially among schoolchildren from the Albaha province in Saudi Arabia. Undeniably, the majority of the quality of life dental research has focused on older age groups based on the assumption that those age groups would have greater oral disease experiences and quality of life impacts (Grath, Bedi, & Gilthorpe, 2000). Consequently, the present study aimed to examine the relationships between the OHRQoL and the different oral diseases, with a focus on a younger age group, particularly 12‐ to 15 year‐old Saudi schoolchildren.

Regardless of the methodological disparities between the OHRQoL measures in the dental literature, there are several explanations for the variations in the oral impact frequencies across different populations and countries. First, different communities have dissimilar social, economic, cultural, and ethnic groups; therefore, the communities may vary with regard to their oral health perceptions and their OHRQoL impacts. Second, population diversity may result in different attitudes and values towards oral health, which could considerably affect the decisions of those different populations with regard to their reported oral impacts. For instance, subjects who place little value on their periodontal conditions are perhaps less likely to report that they are emotionally concerned about their oral health and vice versa.

The findings of this study revealed that the overall prevalence of the oral impacts on the daily activities was 75.1%. This percentage is very significant and is approximately twice the value seen in other studies of similar age groups (Hongxing et al., 2014; Yusuf et al., 2006). This may be due to the differences in the age groups, oral disease levels, cultures, and locations of the study subjects. Nevertheless, our study showed that the most frequently seen oral impact was on eating and enjoying food (54.4%). This is consistent with other Child‐OIDP findings from the United Kingdom, Brazil, and China (Castro et al., 2008; Hongxing et al., 2014; Yusuf et al., 2006). However, further research may be warranted, with a focus on explaining why the oral impact on eating and enjoying food was the most commonly reported impact across these different countries.

The oral impacts were quite prevalent among the Saudi schoolchildren in this study; however, the majority of those subjects describing impacts reported moderate or minor severities of those impacts (Table 4). These results are similar to those of other studies with comparable age groups (Nagarajappa, Batra, Sanadhya, Daryani, & Ramesh, 2015; Nurelhuda et al., 2010). Moreover, the current study revealed that approximately three quarters of the subjects described oral impacts on one or more performance variables, with the most commonly reported impacts on eating and enjoying food (54.4%), cleaning teeth (46.7%), and smiling (11.5%). These findings are in accordance with the results of several previous Child‐OIDP studies in Morocco, Brazil, and India (Athira et al., 2015; Lazrak, Bourzgui, Serhier, Diouny, & Othmani, 2017b; Nagarajappa et al., 2015). The reasons for these commonly reported oral impacts may first be attributed to the importance of one's facial appearance, because it can have significant psychosocial effects on one's life and interpersonal relationships (John et al., 2014). Second, these commonly reported impacts seem to be related to the main functions of the mouth (i.e., mastication), and this may reinforce the significance of reporting such impacts (Hama et al., 2017).

The results of this study pointed out significant associations (*p* < .05) between the different clinical indicators and the oral impacts on the daily performance factors. For example, bleeding gums was significantly associated with the cleaning teeth and smiling performance factors (*OR* = 7.9 and *OR* = 3.1, respectively). The reason for this strong association could be explained by the observation of bleeding gums in the majority of the subjects (81%). These findings are consistent with those of other studies conducted in Brazil (Pulache, Abanto, Oliveira, Bönecker, & Porras, 2016). Likewise, the present study revealed that toothaches were more commonly associated with eating and enjoying food (*OR* = 11.5), speaking (*OR* = 7.33), smiling (*OR* = 3.8), relaxing and sleeping (*OR* = 5.7), and social work (*OR* = 5.2). This may refer to the pain associated with a high prevalence of dental decay among the population studied (79%) (Mashoto, Åstrøm, David, & Masalu, 2009); however, these results are similar to those of other Child‐OIDP studies conducted in Thailand and Sudan (Krisdapong, Sheiham, & Tsakos, 2009; Nurelhuda et al., 2010). Nevertheless, there is no guarantee that oral disease will negatively influence the personal perceptions of one's quality of life and well‐being. When it does, its impact is affected by several factors, including the nature of the oral disease, the subject's preferences and expectations, and their psychological, social, and financial resources (Tsakos, Steele, Marcenes, Walls, & Sheiham, 2006).

The present study did have some limitations. First, the study sample did not include female schoolchildren. This was mainly due to the cultural and traditional challenges encountered when attempting to access females of this age group in their schools, because the researchers who conducted this study were men. Unfortunately, this may have affected the generalisability of the findings of this study. However, this study does provide insight into OHRQoL evaluations and the impacts of the oral diseases of the population from the Albaha province in Saudi Arabia. Second, the study design was cross sectional, which may pose issues with regard to testing the hypothesis, particularly because the evaluations of the risk factors and outcomes were conducted at the same time. Fortunately, this specific issue did not seem to influence the study findings, because the study aim was to explore the associations between the OHRQoL and the different oral diseases among Saudi schoolchildren, rather than evaluating the causal relationships.

## 5 CONCLUSIONS

The prevalence and severity of the oral impacts on the children's daily activities were significantly high. Additionally, associations between several of the clinical oral health measures and the perceived sociodental impacts were detected in the study population. Almost three quarters of the subjects described oral impacts on one or more performance factors, with the most commonly reported impacts on eating and enjoying food (54.4%), cleaning teeth (46.7%), and smiling (11.5%). Further research focusing on developing strategies for oral health promotion and education, in addition to enhancing the knowledge of existing dental treatments and the provision of dental services in this community, is encouraged.

## CONFLICT OF INTEREST

The authors declare no conflicts of interest regarding the authorship and/or publication of this article.

## AUTHOR CONTRIBUTIONS

A. A. made substantial contributions to conception and design of this study and to collecting, analysing, and interpreting of data, whereas E. A. made significant contribution to collecting and analysing of data. M. A. contributed significantly to data analysis and interpretation besides revising the manuscript critically for important intellectual content. Nevertheless, all authors A. A., E. A., and M. A. contributed to drafting the manuscript, providing final approval of the version to be published, and ensuring that questions related to the accuracy or integrity of any part of this study are appropriately investigated and resolved.

## ETHICAL CONSIDERATIONS

Ethical approval was obtained from the Planning, Research and Studies Department at the Saudi Ministry of Education, Albaha branch, Saudi Arabia (approval number: 39195280).
